# Effect of Helpers Stay Quit Online Training on Preventing Smoking Relapse and Personal Networks: Protocol for a Pragmatic Randomized Controlled Trial and Embedded Mixed Methods Personal Network Study

**DOI:** 10.2196/82140

**Published:** 2026-01-27

**Authors:** Myra Muramoto, Allison Hopkins, Christopher McCarty, Alicia Allen, L Miriam Dickinson, Timothy Connolly, Janet Spradley, Jun Ying

**Affiliations:** 1 Department of Family Medicine, School of Medicine, University of Colorado, Rm 3613A, 12631 E. 17th Ave, Aurora, CO, 80045, United States, 1 303-724-9375, 1 303-724-9746; 2 Department of Anthropology, Texas A&M University, College Station, TX, United States; 3 Department of Anthropology, College of Liberal Arts and Sciences, University of Florida, Gainesville, FL, United States; 4 Department of Pediatrics, National Center for Opioid Research and Clinical Efficacy, Arkansas Children’s Research Institute, University of Arkansas for Medical Sciences, Little Rock, AR, United States

**Keywords:** smoking cessation, tobacco cessation, relapse prevention, online training, cessation training, brief intervention, behavioral intervention, mHealth, social network, randomized controlled trial, pragmatic trial, mixed methods

## Abstract

**Background:**

Despite major gains in smoking cessation treatment, over half of those who recently quit will relapse within one year. Two systematic reviews of relapse prevention studies reached differing conclusions on the effectiveness of behavioral interventions. Existing relapse prevention evidence is limited by study designs, methodology, and conceptual approaches to behavioral interventions. Personal networks exert powerful effects on initiating and maintaining smoking behavior and can facilitate maintaining abstinence or trigger relapse. To date, relapse prevention interventions have focused on those who are newly abstinent (“abstainers”) and have not attempted to influence the abstainer’s personal network. The Helpers Stay Quit (Helpers Stay Quit) online training is a conceptually novel “help others” intervention to increase abstainers’ public identification as a nonsmoker and their ability to influence those in their personal network to also quit smoking—thereby creating a personal network social environment supportive of long-term abstinence.

**Objective:**

This study is a 2-arm, pragmatic randomized controlled trial (pRCT) testing the hypothesis that quitline abstainers receiving the Helpers Stay Quit intervention will have higher 30-day and 7-day point prevalence of abstinence than those receiving quitline follow-up usual care, and that outcomes may be mediated by characteristics of abstainers’ personal networks. The embedded mixed methods study examines the effects of the Helpers Stay Quit intervention on the abstainers’ personal network interactions related to smoking and smoking cessation.

**Methods:**

The study design is a 2-group pRCT (N=940) comparing the Helpers Stay Quit online training intervention with a quitline usual care condition. Baseline, 3-, 6-, and 12-month surveys collect data on cognitive and emotional factors potentially influencing relapse. Text messages survey tobacco use status and participants’ use of the Helpers Stay Quit training content. The composition and structure of participants’ personal networks are assessed at baseline and 12 months. We interviewed 60 participants (both relapsed and abstinent) at differing intervals in the last 6 months of study participation to qualitatively assess personal network influences on relapse or abstinence.

**Results:**

A total of 9 state quitlines are participating by referring potentially eligible clients for screening and potential enrollment. Recruitment began in December 2022. Enrollment of 940 participants was completed in September 2025. When the manuscript was submitted, as of August 31, 2025, 337 participants (65%) had completed the study with 12-month follow-up surveys.

**Conclusions:**

This pRCT tests whether exposure to the Helpers Stay Quit intervention decreases relapse rates of newly abstinent smokers enrolled in state quitline coaching treatment. The embedded personal network mixed methods study is designed to examine the characteristics of a newly abstinent smoker’s personal network that may influence relapse and the potential spread of cessation-related information and behaviors through their personal network. The study’s design and measures will provide insights into the influences of personal networks and the cognitive and emotional factors impacting the likelihood of relapse or maintaining abstinence in those who are newly abstinent.

## Introduction

### Background

Effective tobacco dependence treatments are more numerous and accessible than ever before [1,2]. However, tobacco use remains the leading preventable cause of premature morbidity and mortality in the United States. In addition, cessation treatments are severely underused [3-5], due to both knowledge and attitudinal barriers (eg, low awareness of and misinformation about cessation medications’ effectiveness, safety and proper use, and negative attitudes about seeking counseling or other behavioral support for quitting) [6-9]. Despite major gains in the use of smoking cessation treatment, over half of those who have recently quit will relapse within the first year. In an analysis of prospective data from a large representative sample of the general population of the United States, the risk of relapse is more than 50% [10] for abstainers with less than 12 months of abstinence. Identifying effective relapse prevention strategies will enable more individuals to maintain their abstinence beyond a year and, consequently, prevent tobacco-related morbidity and mortality.

Published research on smoking relapse prevention has limitations in methodological design and intervention approaches. Two systematic reviews of relapse prevention interventions reached differing conclusions regarding the efficacy of behavioral interventions [11,12]. Agboola et al [11] concluded minimal self-help intervention was effective. Livingstone-Banks et al [13] concluded with only “moderate certainty” that behavioral interventions were not effective, with the caveat that “further research is likely to have an important impact on our confidence,” [13] and a recommendation that future relapse prevention research examine alternative behavioral approaches [13]. A critique of extant research cited methodological weaknesses and limited conceptual approaches to treatment [13]. Different approaches to both studies of relapse prevention and to the interventions themselves are needed to advance the long-term understanding and outcomes of smoking relapse prevention. Livingstone-Banks et al recommended randomization of smokers who were already abstinent (“abstainers”) as the strongest design for relapse prevention studies [13]. The protocol for a pragmatic randomized control trial (pRCT) we present here addresses both methodological and treatment approach limitations of extant research.

### Conceptual Basis for the Helpers Stay Quit Intervention

Conceptualizing relapse as a dynamic process is the organizing framework [14] guiding the overall Helpers Stay Quit intervention and our evaluation approach. Within this working model, relapse susceptibility or “Relapse Proneness” [15] shifts as a function of physiological, environmental, and cognitive components, each independently contributing to relapse risk at various points in the continuum from quit attempt to long-term abstinence. Relapse proneness results from the interplay of 3 forces—2 of which are cornerstones of relapse research: physical withdrawal symptoms and triggers. The third, more speculative force—“cessation fatigue”—is a latent construct operationalized by declines in motivation, expectancy for success, coping attempts, self-efficacy, and self-control resources [14]. It has been suggested that exploration of “cessation fatigue” may illuminate new possible mechanisms related to relapse [15-17]. To address the dynamic and complex nature of the relapse process and to guide the approach for the proposed pRCT, the conceptual model for the intervention and study uses an integration of interpersonal [18,19], social network [20], social support [21], behavioral theories and concepts and stress and coping theories [22], and Helper Therapy Principle [23] to examine the interrelatedness, time sensitivity, and potential feedback loops related to relapse outcomes [16]. Helpers Stay Quit is novel in its integration of the above theories into an intervention model that is presented as helping others and teaches trainees how to offer a “helping conversation” to help others quit.

### Personal Networks and Smoking Behavior

Personal networks exert powerful influences on smoking and quitting behavior [24]. Efforts to increase the use of cessation aids have focused almost exclusively on the smoker [25,26]. Personal networks, wherein abstainers (“egos”) are in direct contact with members of their personal network (“alters”), warrant exploration as an avenue for relapse intervention. Personal networks can provide positive feedback and support for abstinence. Importantly, personal networks can also be sources of powerful cues to smoke and ready access to cigarettes—directly impacting relapse risk [27-31]. Personal networks may be an important part of the relapse process, but little is known about how relationships and exchanges of knowledge and information between people in the network feed back into the cessation context. Cessation behavior transmission can occur within three degrees of separation from the original quitter [32], suggesting network-based interventions may be beneficial for initiating quit attempts beyond the abstainer’s personal network. In previous work, 89% of general community member participants (n=906) were motivated to enroll in our study to learn tobacco cessation skills to help a friend or family member quit [33]. Furthermore, the majority of participants (up to 80%‐86%) [34,35] reported taking action to help others quit, including offering information and referrals [35,36] primarily to persons in their personal network [35,36].

### Preliminary Evidence Supporting Helpers Stay Quit for Relapse Prevention

We conducted a single-group, observational, pilot feasibility study of Helpers Stay Quit, with an embedded personal network study (n=104), to assess design, methods, and procedures in preparation for the current pRCT and personal network study protocol presented in this paper. Participants were cigarette smokers enrolled in Arizona’s state quitline coaching service contacted for routine follow-up approximately 14‐30 days after their quit date and reporting abstinence from smoking (not even a puff) for at least 14 days. Eligible participants were adults, able to speak and read English, had access to a computer to take the online Helpers Stay Quit training, had a mobile phone to receive and send text messages, agreed to participate in two personal network interviews, and consented to share their quitline client data with the research team. Participants completed online questionnaires at baseline, 3 months, and 6 months, querying smoking status and other behavioral measures related to smoking and cessation. Monthly text message surveys queried smoking status and offering of helping conversations. Personal network telephone interviews were conducted at baseline and 6 months.

We used propensity score matching to compare the quitline clientele with the feasibility study sample at 6‐7 months. Matches were drawn from deidentified data from the quitline’s complete participant pool for the 2-year period corresponding to the study in a ratio of 4 to 1. Variables for matching included age (>50 years vs 18‐50 years), sex (male vs female—no nonbinary participants were enrolled), race and ethnicity (non-Hispanic White, Hispanic, and non-White non-Hispanic), Fagerström score (>5 vs 1‐6), and presence of a chronic condition (any vs none). Study results showed Helpers Stay Quit participants reporting higher 30-day abstinence than nonparticipants (86% vs 36%; difference 49%, 95% CI 40%-59%; *P*<.001) [37].

Participants in the longitudinal embedded personal network study (egos) completed a personal network telephone interview in which they named members of their personal network with whom they had at least monthly contact (alters). With each monthly text message survey, egos who reported offering a helping conversation were given a link to an online survey that could be passed to an alter who could respond anonymously. The alter survey queried smoking status, content of the helping conversation, and the alter’s reaction to the helping conversation. Results showed that a participant’s behavior to help others quit extended to their personal networks, where 89% of abstainers offered helping conversations, and 86% abstained from smoking for the 6-month study duration (n=64) [38]. Abstaining individuals significantly increased the number of nonfamily members (mean difference [MD] 1.41; *P*<.001) and smokers in their networks (MD 0.77; *P*=.006) at follow-up. This change provided participants with more opportunities to carry out helping conversations. After receiving a helping conversation, alters reported positive changes in cessation-related behavior. These findings further support the potential of personal network behavioral interventions, like Helpers Stay Quit, to facilitate smoking relapse prevention.

### Research Objectives

This study is designed to assess the effect of Helpers Stay Quit training on the proportion and duration of participants maintaining abstinence or relapsing over time and on participants’ personal network structure and interactions related to smoking and smoking cessation. Primary and secondary research questions are as described in study-specific aims (Textbox 1).

Textbox 1. **The Helpers Stay Quit study-specific aims.**

- Aim 1: Using a 2-group, pragmatic randomized, controlled trial (pRCT; N=940) in which abstinent smokers (2-6 weeks abstinent) are randomized to the Helpers Stay Quit intervention or quitline follow-up usual care, to evaluate the effectiveness of Helpers Stay Quit on:
  - Smoking status (30-day and 7-day point prevalence abstinence) at 6- and 12-month intervals (primary outcome).
  - The number, timing, and duration of participants’ lapses and relapses to smoking (secondary outcome).
  - Rates of participants’ self-reported delivery of helping conversations to other tobacco users (secondary outcome).
- Aim 2: embed a mixed methods social network study into the pRCT to examine the Helpers Stay Quit effects on:
  - Composition and structure of participants’ personal networks.
  - Characteristics of participants’ personal networks over the course of the study (eg, proportion of smokers in personal network).
  - Spread of smoking cessation-related information, assistance, and behaviors from participants to others in their personal networks.
  - Recipients of helping conversations report about smoking-related interactions and information exchange with participants and associated cessation-related behavior change.
- Aim 3: using personal network metrics derived from the personal network study (eg, size, composition, and structure), conduct mediational analyses of personal network effects on the primary and secondary study outcomes in Aim 1.

## Methods

### Study Design

**Figure 1.**
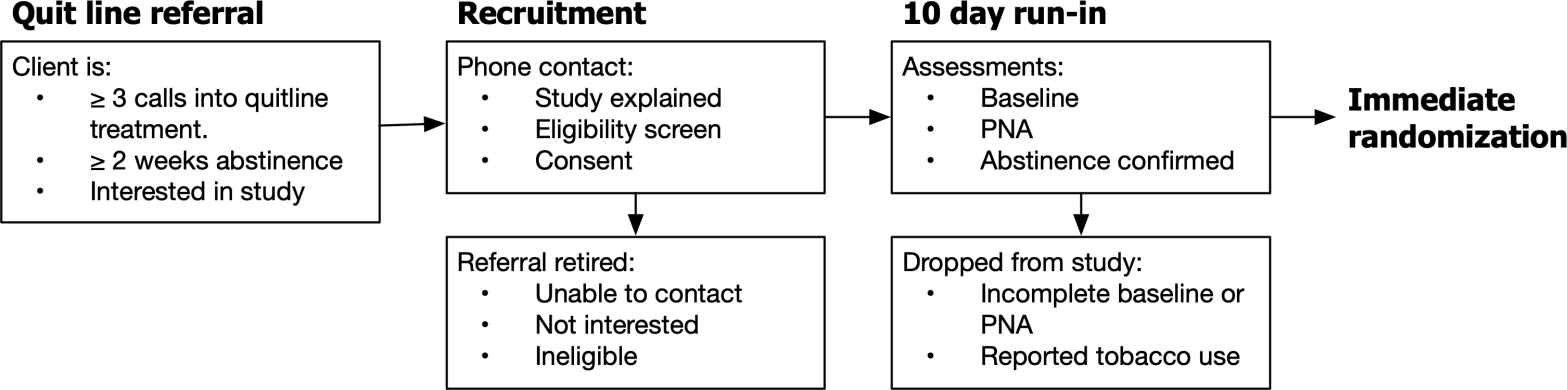
Timeline for recruitment, screening, consent, and prerandomization run-in for the helpers stay quit pragmatic randomized controlled trial and embedded personal network assessment (PNA).

**Figure 2.**
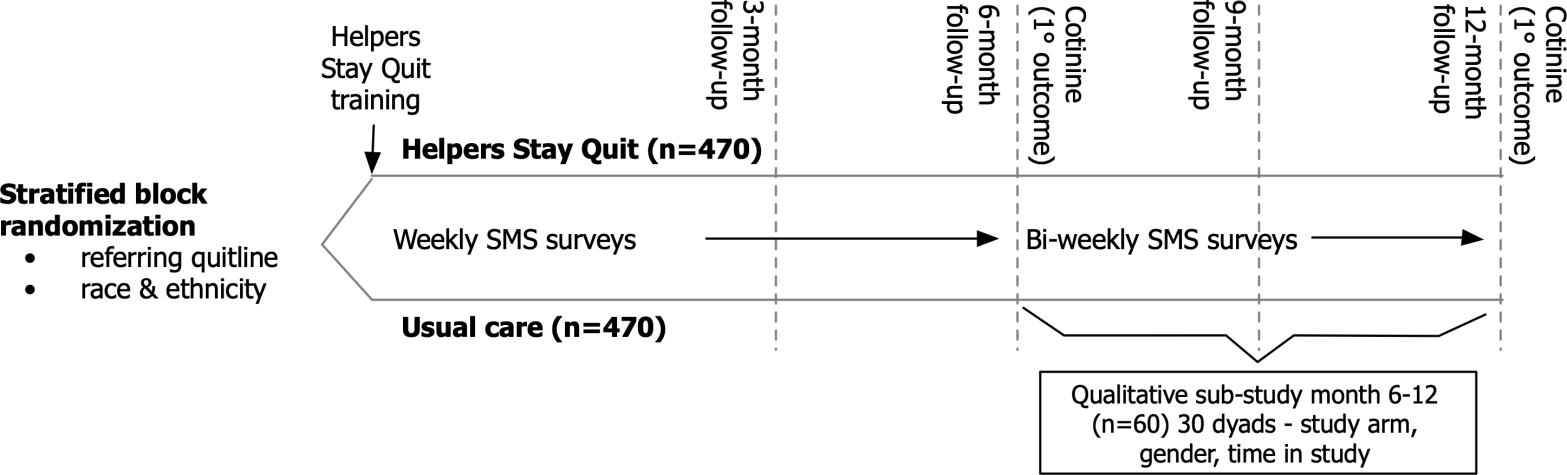
Postrandomization study timeline and data collection schedule for the Helpers Stay Quit (HSQ) pragmatic randomized controlled trial.

This is a 2-group, parallel pRCT with embedded personal network study comparing: (1) Intervention: Helpers Stay Quit training, and (2) control: quitline postabstinence follow-up usual care. The primary outcome is smoking status at 6 and 12 months. The purpose of the pRCT is to assess if a novel behavioral intervention (Helpers Stay Quit) overall has any effect on relapse in newly abstinent smokers following standard quitline treatment compared to quitline usual care, in real-world conditions of serving state quitline client populations. Relapse and the number of helping conversations will be assessed using a text survey weekly for the first 6 months and biweekly for months 7‐12. The prestudy quitline treatment, quitline referral, study recruitment, screening, consent, and prerandomization and run-in period are depicted in Figure 1. The postrandomization study intervention exposure, assessment intervals, and qualitative interviews are depicted in Figure 2.

### Ethical Considerations

The Helpers Stay Quit study was approved by all participating institutions’ institutional review boards (IRBs), including the Combined Multiple Institution Review Board of the University of Colorado (21‐4624), Arizona Department of Health Services (HSRB # 23‐0016), Kentucky Cabinet for Health and Family Services IRB (CHFS-IRB-DPH-FY23-35), Massachusetts Department of Public Health (MDPH IRB 00000701), Ohio Department of Health Human Subjects IRB (2023‐21), Pennsylvania Department of Health IRB (2022‐065), and Utah Department of Health and Human Services IRB (686).

The IRBs for Texas A&M University, University of Florida, Kansas Department of Health and Environment, Michigan Department of Health and Human Services, and the Vermont Agency of Human Services each determined that the Helpers Stay Quit study could be implemented without additional IRB review.

Informed consent was conducted by telephone; participants could see the consent form on a screen, could electronically sign the form, and were emailed a copy of their signed consent. As part of the informed consent process, participants were read paragraphs from the consent form as shown in Multimedia Appendix 1.

Study participants’ confidentiality is protected by the following measures: all study participants are assigned a numerical study ID. The linking file that contains both study ID and participant-identifying information (name, address, telephone number, and email) is stored on a secure server in a physically secured server room. The linking file is only accessible by research staff employed by the University of Colorado. Data files shared with co-investigators, statisticians, and analysts are deidentified.

Participants completing every protocol activity and qualifying for every incentive could receive a total of US $419. A breakdown of the study protocol items and their compensation rates is as follows: baseline (US $20), social network assessment 1 (US $25), training (US $40), incentive for completing training within two weeks (US $10), 3-month survey (US $20), 6-month survey (US $20), 9-month survey (US $20), 12-month survey (US $20), bonus for completing all 4 surveys (US $5), social network assessment 2 (US $25), qualitative personal network interview (US $30), weekly text survey (24 total; US $4 each), biweekly text survey (12 total; US $4 each), 6-month follow-up specimen completion incentive (US $15), 12-month follow-up specimen completion incentive (US $15), and maximum completion bonus for completing all study activities (US $10).

### Participant Recruitment, Eligibility, Enrollment, and Randomization

#### Recruitment

Participants are referred from state quitlines based on a prescreening algorithm programmed into the coaching platform that identifies potentially eligible quitline callers self-reporting abstinence during their third, fourth, or fifth coaching call, and interested in being referred to the study. Upon referral from the quitlines, study staff contact participants via telephone to screen for initial study eligibility, provide the participants with a detailed explanation of the study’s purpose and procedures, and obtain electronic informed consent from those who are eligible and wish to proceed to the prerandomization run-in period.

##### Eligibility Criteria

The eligibility criteria are shown in Textbox 2.

Textbox 2. **Eligibility criteria.**

Inclusion criteria:

- Participants are aged 18 years and older.
- Abstinent from smoking for between 14 and 60 days per self-report.
- Primary tobacco use is cigarettes.
- Has access to the internet via computer or mobile device.
- Self-described proficiency with English.
- Willing and able to send and receive weekly text messages using personal mobile phone.
- Will allow the quitline to share its client data with the research team.
- Willing to complete online surveys at baseline, 3, 6, 9, and 12 months.
- Willing to self-collect dried blood spot for biological confirmation of smoking status and send back to the research team.
- If assigned to the Helpers Stay Quit condition, willing to complete the training within 14 days.
- If selected, willing to participate in a qualitative interview.
- Willing to forego any other training for tobacco cessation intervention or support (ie, to become a cessation counselor or facilitator or support person, eg, “quit buddy”) for the duration of their study enrollment.

Exclusion criteria:

- Any previous exposure to Helpers Stay Quit or other cessation training in the previous 2 years.
- Relapse within 10 days (run-in phase) before enrollment and randomization.
- Participants are not precluded from continuing their personal tobacco cessation activities with quitline or other services or aids (eg, support groups, medication, etc).
- Concomitant use of cessation aids is tracked while participants are enrolled in the study via online surveys (baseline, 3, 6, 9, and 12 months, and weekly or biweekly text surveys).

### Prerandomization Run-in/Baseline Measures

A major strength of the Helpers Stay Quit study design is the clear separation of quitline cessation treatment from the Helpers relapse prevention intervention. This separation is maintained by requiring that all participants attest to their continued abstinence immediately before randomization. After initial study eligibility screening, consented participants have up to 10 days to complete study run-in baseline measures, which include a survey of cognitive and emotional measures (Table 1) and a personal network assessment (PNA). Those who fail to complete run-in baseline procedures are not randomized into the study. Immediately before randomization, participants attest to their continued abstinence (7-day point prevalence; Figure 1).

**Table 1.** Data collection schedule for the Helpers Stay Quit study pragmatic randomized controlled trial.

Data collected	Baseline	Training pre-post	Weeks 1‐24	3-month follow-up	6-month follow-up	Weeks 25‐52	9-month follow-up	12-month follow-up	Dyad qualitative interviews, months 5‐12
Tobacco use or abstinence (1° outcome)	✓		✓	✓	✓	✓	✓	✓	
Cotinine					✓			✓	
Helping conversations (2° outcome)			✓	✓	✓	✓	✓	✓	
Helping conversation content				✓	✓		✓	✓	
Personal network assessment	✓							✓	
Cessation knowledge		✓							
Cessation fatigue	✓			✓	✓		✓	✓	
Abstinence Related Motivational Engagement-Short Form	✓			✓	✓		✓	✓	
Smoker or quitter identity	✓			✓	✓		✓	✓	
Smoker group identity	✓			✓	✓		✓	✓	
Residual attraction or vulnerability to smoking	✓			✓	✓		✓	✓	
Situational smoking abstinence self-efficacy	✓			✓	✓		✓	✓	
Proactive Coping Inventory	✓			✓	✓		✓	✓	
Patient Health Questionnaire-4	✓			✓	✓		✓	✓	
Qualitative aspects of network change, continuity									✓

### Randomization

Participants are randomized using stratified block randomization to ensure balance between study arms within key subgroups. Randomization is stratified by quitline and race and ethnicity (categorized as non-Hispanic White, Hispanic, non-Hispanic Black, and other). Within each stratum, participants are assigned to intervention or control groups in random block sizes of 4 and 6. The use of variable block sizes minimizes the predictability of treatment allocation while maintaining approximate balance in group assignments throughout the enrollment process. Randomization is implemented using a computer-generated randomization schedule prepared by the study biostatistician and concealed from study personnel involved in participant recruitment and assessment.

### Intervention and Comparison Conditions

#### Helpers Stay Quit Intervention

The Helpers Stay Quit intervention is a web-based tobacco cessation brief intervention training designed specifically for lay community members interested in helping someone in their lives quit tobacco. The multimedia, interactive, web-based training emphasizes a tobacco user–centered, nonconfrontational approach to encouraging others to quit. Helpers Stay Quit trainees (ie, “Helpers”) learn how to offer a 4-step “helping conversation” to encourage quitting and use of evidence-based cessation aids (eg, cessation medications, and quitlines), and to offer referrals to quitlines and reliable information sources (eg, pharmacists, National Cancer Institute’s SmokeFree website). The 4 steps of a helping conversation parallel the 4 core training modules of Helpers Stay Quit (awareness, understanding, helping, and relating), incorporating key components of the Public Health Service guideline’s recommended 5As [39].

A key learning objective of Helpers Stay Quit training is for trainees to learn how to manage their own expectations for the process and outcome of a helping conversation. For example, they cannot “make” anyone quit, but they can offer nonjudgmental encouragement and support and information about effective cessation aids. Helpers are taught to avoid nagging, confrontation, or pushing anyone to quit. The helping conversation focuses on encouraging behavior change that is aligned with the tobacco user’s current willingness and readiness to take any action toward quitting. Helpers Stay Quit includes content on evidence-based cessation aids and sources of reliable information about quitting. The training includes video role-plays of helping conversations and testimonials of current and former tobacco users and “concerned others” to illustrate core concepts. Learning activities prompt personal reflection and require application of new knowledge through scenario-based quizzes and decision-testing (eg, tobacco use, benefits of quitting) and skills (eg, recognition of appropriate use of communication techniques, identifying optimal or suboptimal responses to helping conversation interactions). Participants also have access to downloadable “Helpers tools,” which are simple brochures on topics such as “Thinking of Quitting” and “Benefits of Quitting.” The training takes approximately 2.5 hours to complete and includes a pre- and posttest and a downloadable certificate for successful training completion.

#### Quitline Usual Care Comparison

Quitline posttreatment usual care for our participating quitlines is to contact clients for follow-up assessment of abstinence at 7 months after enrollment in services, regardless of the number of calls completed. If the client has relapsed, the quitline attempts to reengage the client in cessation services. The participating quitlines each have a website that accompanies and complements their telephonic services. The websites contain information about tobacco use, tobacco cessation, descriptions of telephonic quit coaching services, and how to access quit coaching and cessation medications offered by the quitline. None of the quitline websites offer information specific to how to help others quit tobacco.

### Measures and Data Collection for Pragmatic Randomized Controlled Trial (pRCT)

#### Quitline Client Data

Participants consent for their quitline data to be shared with the study team including demographics (age, gender, and race and ethnicity), education, income level, presence or absence of behavioral health or chronic disease conditions, zip code, detailed tobacco use history and current use, time to first cigarette, use of electronic nicotine delivery systems, cessation medications provided and used, and number of quitline coaching calls completed.

#### Relapse Status (Primary Outcome) and Helping Conversations (Secondary Outcome)

Smoking or other tobacco use, electronic nicotine delivery device use, use of cessation aids, and helping conversations are assessed by online surveys at baseline, 3, 6, and 12 months, and by weekly text message surveys for months 1‐6, then biweekly text surveys for months 7‐12. Relapse is defined as (1) any smoking on 7 consecutive days or (2) smoking at least once each week over 2 consecutive weeks. Multiple relapses are defined as relapsing, reporting being quit (7-day point prevalence), and then subsequently reporting smoking again. Offers of helping conversations are assessed by baseline survey and weekly text message surveys (months 1‐6), then biweekly text surveys (months 7‐12). A 5-point Likert scale queries confidence in helping conversations, for example, talking to others about quitting smoking. Content of typical helping conversation (eg, setting quit date, quit plan, cessation medication, quitline referrals, and avoiding relapse) is queried via a yes or no checklist at baseline, 3, 6, 9, and 12 months (Table 1).

#### Cognitive or Emotional Measures

A dynamic model of smoking relapse is the organizing framework guiding the overall intervention and evaluation approach [14]. Selected measures evaluate possible factors in “cessation fatigue,” a latent construct proposed to contribute to “relapse proneness” and operationalized by declines in motivation, expectancy for success, coping attempts, self-efficacy, and self-control resources [14]. To explore the concept of “cessation fatigue” [16], the following patient-reported outcome measures are collected at baseline, 3, 6, 9, and 12 months post study enrollment: cessation fatigue [17], Abstinence Related Motivational Engagement – Short Form [40], smoker versus quitter identity [41], smoker group identity [41], residual attraction and vulnerability to smoking [42], situational smoking abstinence self-efficacy [43], Proactive Coping Inventory [44,45]; and Patient Health Questionnaire-4 for anxiety and depression [46-48] (see Table 1).

#### Biochemical Confirmation of Abstinence

Participants who self-report abstinence on 6- and 12-month surveys are mailed a kit for self-collection of the dried blood spot. Participants are trained on self-collection of dried blood spots using an online video and instructional booklet [49]. Dried blood spot samples will be analyzed for cotinine to confirm self-report, following previously validated approaches [50] (Table 1).

### Measures and Data Collection for Embedded Mixed Methods Personal Network Study

#### Overview

Personal network recruitment, screening, and inclusion and exclusion criteria are the same as for the main study. All main study participants (egos) complete two online personal network assessments, one at baseline and the other at 12 months post baseline. Personal network assessment questions address network composition and structure and are administered using REDCap (Research Electronic Data Capture; Vanderbilt University) [51-54]. Each participant (ego) is asked to name 25 individuals (alters) with whom they have had any kind of contact over the past six months [55,56]. Egos are then asked questions about each alter to capture the compositional variables, such as the proportion of individuals who smoke in their personal network and the social support and influence for abstinence. Personal network structure is defined by asking the participant (ego) whether each unique pair of alters talks to each other independently of ego [52].

#### Alter Surveys

We use respondent-driven sampling [57-62] to survey smoking alters who receive a helping conversation from the ego to measure the spread of smoking cessation information and behaviors. With each weekly (months 1‐6) or biweekly (months 7‐12) period, egos also receive a text message link to an anonymous online survey (alter survey) that can be passed on to alters for completion. Alter surveys query smoking status, reaction to the helping conversation, context and content of the helping conversation, and if the alter has shared helping conversation information with others in their own personal network.

#### Personal Network Qualitative Interviews

A subsample (n=60) was selected for qualitative interviews about relapse or abstinence and the qualitative aspects of personal network continuity or change [63,64], interactions with members of personal network, exchange of information, assistance, or goods related to smoking cessation. Participants who had completed at least 5 months of the study were selected in 30 dyads (a dyad is one relapser paired with a like abstainer or maintainer from the same study arm, matched on gender). The 15 relapser or maintainer dyads from each arm are matched on the approximate number of weeks participating in the study. Network visualizations on connectedness, alter centrality, closeness, interaction, demographics, and smoking status are generated using the R software (R Foundation for Statistical Computing) package *ggraph* and shown to participants to facilitate discussion in qualitative interviews.

### Sample Size, Power Estimates, and Analysis

#### Power and Sample Size for Pragmatic Randomized Controlled Trial (pRCT)

A sample size of 940 participants would yield 80% power to detect a difference in smoking cessation rates between arms of 10%, assuming a conservative control arm rate of 0.25 [65], 30% dropout, and a noncontinuity corrected *χ*^2^ test. This difference is smaller than that found in the preceding pilot feasibility study (difference=46%); this effect size was chosen as a clinically significant difference with public health implications and is feasible, given the pilot results. This sample size also yields 90% power to detect standardized effect sizes for continuous secondary outcomes of Cohen *d*=0.26, a small-to-medium effect size, and considered to be a good estimate for a minimum important difference for patient-reported outcomes.

#### Personal Network Study Sample Size

To measure change in personal networks at two times, 940 participants (470 in each arm) will be sufficient. Although there are few longitudinal personal network studies among those with substance use disorders or those who have recently quit using as models [55], studies using dynamic network analyses to estimate the impact of life events on an individual’s social context typically include between 33‐250 participants [66-72]. Informed by the previous feasibility study, the present personal network study features an increased sample size, a lengthened interval between the first and second personal network assessment to 1 year, and broadened the personal network name generator question to capture both strong ties and weak ties. The qualitative sample (n=60) is theory-driven; sufficient for coverage of basic consensus and range of variability of critical themes [73].

#### Analytic Plan

##### General Quantitative Approaches

For this pRCT, descriptive statistics (mean, SD, and frequencies) will be computed for baseline participant characteristics. Participant-level covariates will be screened in bivariate analyses and included in multivariate analysis if they are related to the outcome at *P*<.2, are associated with dropout, or used in the randomization procedure. Before beginning the analyses described below to address study hypotheses, we will compare baseline characteristics of participants who drop out to participants with complete data to assess the missingness mechanism (missing completely at random [MCAR], missing at random [MAR], and missing not at random [MNAR]) and generalizability of our results [74-76]. If missingness is ignorable (MCAR and MAR), we will use likelihood-based methods that use all available data, adjusting for covariates that are associated with dropout. If missingness is nonignorable, we will use pattern mixture models. Sensitivity analyses with multiple imputation will be undertaken if missing data rates for the primary outcome are greater than 10% at any follow-up time used in the analysis.

##### Aim 1: Abstinence and Relapse

Abstinence rates at 6- and 12-months postrandomization and biochemically confirmed abstinence at 6- and 12-months postrandomization will be compared between arms using multivariable logistic regression for each outcome (6- or 12-months). Independent variables will include intervention status, strata (race and ethnicity), quitline, and covariates (as described above). We will compare time to first relapse using Cox models and Kaplan-Meier curves. The number of relapses and helping conversations will be compared between study arms using Poisson regression, with the zero-inflated Poisson or negative binomial model used if appropriate. We will carry out secondary analyses using general linear mixed models to examine change in cognitive and emotional measures of participant-reported outcomes (described above in the General Quantitative Approaches section) for participants randomized to receive Helpers Stay Quit compared to participants randomized to the usual care arm.

##### Aim 2: Personal Network Study

We will use quantitative personal network analysis to address Aims 2a, b, and c. Personal network measures consist of compositional variables, which describe who is in the network, and structural variables, which measure how network alters are arranged around the ego. Personal network measures will be obtained at two points, baseline and 12-month follow-up. Compositional variables for each alter include: demographic data (ie, sex and age); relationship to participant (ie, relationship, how close, frequency of interaction, and smoking- and quitting-related support); and smoking-related information (ie, use of tobacco products, previous and current smoking status, and quit attempts). Structural variables will be calculated in R package *egor* based on alter pairs that very likely interact and include: density (ie, the extent to which all possible connections between alters are present), alter centrality measures (ie, to identify the most central individuals in the network), network centralization (ie, the extent to which a network is dominated by one or a few alters), and measures of subgroups (ie, to identify cohesive or disconnected subgroups). Additionally, some combined compositional and structural variables will be calculated with smoking status and alter centrality measures.

Descriptive statistics will be run on all compositional and structural personal network variables at both time points. We will assess using analysis of variance differences in network measures between the Helpers Stay Quit (vs usual care completers, completers who carried out helping conversations (vs no helping conversations), and completers who relapsed (vs abstained). Paired samples *t* tests will be used to compare network measures between the two different points.

Qualitative analysis will be used to address Aims 2a, b, and c, along with the quantitative personal network analysis. Participants were shown a series of network visualizations to facilitate discussion during the interview. Interviews are transcribed, using a verbatim transcription protocol, and analyzed using a combination of a priori (theory-based) coding combined with emergent codes to produce themes, which are used to assign higher-level meaning to the data [77-79]. Manual coding using ATLAS.ti (Scientific Software Development GmbH) will be compared to AI coding using natural language processing in R. Both descriptive and interpretive codes will be used in this analysis, with development of additional codes for emergent themes and issues [79].

##### Aim 3: Mediational Analysis

Using data from Aims 1 and 2, we will use multiple mediation models to investigate mechanisms of Helpers Stay Quit. Specifically, we will estimate the direct effects of the intervention on abstinence, as well as the indirect effects that are mediated through the personal networks of participants and longitudinal change in scores collected through surveys. Potential candidates for mediation include the number of helping conversations, change in personal network variables (smoking status, smoking status and degree centrality, smoking status and sex, density, and degree centralization), and change in patient-reported outcome variables from the cognitive and emotional measures. To narrow down the list of potential mediators from the conceptual framework, we will first examine relationships between the intervention arm and the mediator, as well as the mediator and the outcome variable. Variables associated with both the intervention and abstinence, with a *P* value of .1, will be included in this analysis as potential mediators. Variables associated with abstinence but not treatment arm at the 0.1 alpha level will be included as a covariate but not explored further as a mediator. For those who meet the initial criteria for a mediator (associated with both intervention arm and outcome), we will examine direct and indirect effects using causal mediation modeling [80].

## Results

This trial was funded in 2021 and is currently ongoing. Initial study start-up was delayed when the first author, MM, moved to the University of Colorado. Recruitment began in December 2022, and we completed enrollment and randomization of 940 participants on September 15, 2025. As of August 31, 2025, when the paper was submitted, 337 participants (65%) had completed the study with the 12-month follow-up surveys.

## Discussion

### Principal Findings

We describe the protocol for a rigorous evaluation of a conceptually novel behavioral intervention for smoking relapse prevention with a study design and intervention addressing methodologic weaknesses and limited conceptual approaches to treatment in extant research [81].

Treatment approaches described in the literature have focused on skills-based interventions that encouraged identifying and resolving tempting situations, and minimal interventions using single sessions or written materials [81]. All behavioral interventions had abstainers apply skills to themselves [81]. Methodological limitations included small sample size, lack of clear separation between treatment and relapse prevention interventions, or insufficient detail to permit survival analyses [81]. Randomization of smokers who were already abstinent is recommended as the strongest design [81]. This ongoing pRCT addresses both methodological and treatment approach limitations of extant research, with a clear separation of cessation treatment from relapse prevention by randomly assigning smokers who have quit for at least 14 days. Helpers Stay Quit’s integration of behavioral theories and conceptual models is a conceptually novel approach to behavioral intervention, not previously studied.

Helpers Stay Quit’s “help others” presentation encourages abstainers to help other smokers to quit. Helpers Stay Quit integrates multiple behavioral theories into a novel intervention model that is presented as “help others.” Helpers Stay Quit teaches knowledge and skills to help others to quit tobacco, and thus could potentially increase an abstainer’s ability to influence their personal network in ways supportive of prolonged abstinence, for example, transmitting cessation behavior to smokers in the personal network [32,82]. This approach could potentially reinforce an abstainer’s identity as a nonsmoker and help shift the personal network social environment toward nonsmoking. Smoking-related identities and smoking behavior are reciprocally interrelated [41,83]. By offering helping conversations to others in their personal network, an abstainer publicly declares and reinforces a “quitter” or “non-smoker” social identity (vs smoker identity) within their personal network and to the abstainer themselves, creating a social expectation that favors abstinence and identification as a nonsmoker over time [84]. Engaging in prosocial helping behavior may be an alternate or additional proactive coping strategy for abstainers to deal with stressors potentially impacting relapse [85-87].

Research on the effect of social networks on smoking cessation and relapse among adults in the general population is limited. Most current studies of adult smoking and social networks focus on large online communities of tobacco users engaged in social media related to smoking and smoking cessation, for example, QuitNet [32,88-91], Become an EX [92,93], and Facebook pages with a smoking-cessation theme [94]. Much of the other research on smoking and social networks addresses adolescent or young adult smokers and smoking initiation [95] or pregnant and postpartum women [81]. Stone and colleagues [96] identified two different network approaches used in studies of personal networks and recovery from substance use, and by extension, smoking cessation: network predicting outcome and treatment predicting network change. The network predicting outcome studies are more prevalent and focus on the changes in the smokers’ personal network after cessation in a general population [90], during pregnancy [27], or among adults with serious mental illness [97]. Treatment-predicting network change studies are less common and include Helpers Stay Quit. Helpers Stay Quit is an intervention (“treatment”) intended to facilitate (“predict”) network change through helping conversations. There are several benefits to this approach. First, it allows participants to maintain their network members, which was identified as a factor in Nguyen and colleagues’ [27] study on why women did not stay quit after they gave birth. It also potentially reduces the participants’ contact with people who smoke by assisting their network members in quitting smoking, which was an important factor for those who stayed quit in Bray and colleagues’ study [90]. Finally, it leverages the valuable resource that people who quit smoking become for assisting other network members in quitting [97].

### Strengths and Limitations

The Helpers Stay Quit study’s strengths include a methodologically rigorous design, a pRCT with “real world” participants recruited from multiple state quitlines, and the embedded mixed methods personal network study. The study’s innovation, described below, is another key strength.

Helpers Stay Quit integrates multiple behavioral theories into an intervention model that is innovative both in its theoretical approach and its “help others” presentation. The beneficial effects of helping others on the helpers themselves [23] have been demonstrated in diverse other groups well beyond mental illness and addiction treatment. Yet, the impact of helping others quit on the newly abstinent smokers themselves has not been previously studied. Also novel is the integration of behavioral theories from interpersonal [18,19], social network [20], and social support [21], stress and coping theories [22], and Helper Therapy Principle [23] into a multifaceted intervention addressing relapse proneness within a framework of relapse as a complex and dynamic process. This is one of the first attempts to include the structural and compositional properties of a personal network as a covariate in smoking cessation.

Activating abstainers to offer helping conversations within their personal network could create new avenues of exploration for relapse prevention. This study examines the effect of the Helpers Stay Quit intervention on the structure and composition of abstainers’ personal networks. By measuring personal network changes over time, we can assess if abstainers could potentially reduce their risk of relapse by decreasing the number of smokers in their personal network, thereby decreasing exposure to smoking cues, access to cigarettes from other smokers, potentially shifting their personal network’s normative behaviors toward nonsmoking, and overall creating a social environment that is more conducive to maintaining abstinence [16]. Abstainers may also be able to make changes in their relationships that affect the position of smokers within their networks, such as making smokers less central.

The study’s limitations include the following: the Helpers Stay Quit intervention is currently only available in English, so participants must be proficient in English. The state quitline prescreening and referral algorithm only identifies clients who are abstinent and have completed 3 or more coaching calls, so we will be unable to assess intervention impact on clients who could be abstinent after fewer than 3 calls. The Helpers Stay Quit intervention is an integration of concepts from interpersonal [18,19], social network [20] and social support [21], stress and coping theories [22], and Helper Therapy Principle [23] into a multifaceted intervention model collectively presented to participants as “helping others.” This first trial after our feasibility trial is designed to assess whether Helpers Stay Quit overall has any effect on relapse, not which of the theoretical concepts or behavioral mechanisms has an effect. Thus, we will be unable to discern if there is a differential effect from a particular intervention component. If Helpers Stay Quit overall has a favorable effect on relapse, future studies with different designs and comparator conditions could examine which aspects of the model may be most salient. National Jewish Health operates the quitline for all the participating states, so we will not know if results from the Helpers Stay Quit study would also apply to other states with different quitline vendors. As with any study with inclusion and exclusion criteria, and participant self-selection to enroll, the study sample may differ from the general population of quitline clients who did not qualify or chose not to enroll. The study is recruiting from clients enrolled in state quitline services, and the majority of quitline clients are women. Since the majority of smokers attempting to quit do not engage with quitlines, study findings may not generalize to the general population of smokers.

### Conclusions and Future Work

In summary, this study aims to address current limitations in smoking relapse prevention research using the recommended strongest study design and a conceptually novel approach and presentation of an online behavioral intervention. Should Helpers Stay Quit demonstrate a beneficial effect on smoking relapse, the low marginal cost and scalability of a web-based intervention, and the ease of integration into existing cessation services increase the potential reach. Even small effects can be meaningful on a population level if the intervention can be delivered efficiently at a large scale and low cost. Follow-up papers will report on trial results following the analytic plans described in this paper. Findings from this trial will help advance scientific knowledge on smoking relapse prevention from different perspectives and domains of possible inquiry.

## Supplementary material

10.2196/82140 Multimedia Appendix 1 Consent form paragraphs read to participants as part of the informed consent process.

10.2196/82140 Checklist 1 SPIRIT checklist.

## Abbreviations

IRB: institutional review board
MAR: missing at random
MCAR: missing completely at random
MD: mean difference
MNAR: missing not at random
PNA: personal network assessment
pRCT: pragmatic randomized controlled trial
