# Supplementary material for: Effect of Helpers Stay Quit Online Training on Preventing Smoking Relapse and Personal Networks: Protocol for a Pragmatic Randomized Controlled Trial and Embedded Mixed Methods Personal Network Study
Source: JMIR Res Protoc. 2026 Jan 27;15:e82140. doi: 10.2196/82140 (PMC12840865; doi:10.2196/82140)
Supplement: Multimedia Appendix 1 [file resprot-v15-e82140-s001.docx]

***Consent form paragraphs read to participants as part of the informed consent process***

**“Will I be paid for being in the study? Will I have to pay for anything?**

You will be paid for all the study items you complete. You will not be paid for any items you do not complete. If you complete every item and qualify for every incentive, you could receive a total of $419. The payments will be distributed over the time you are in the study. You will have access to an online website that will show your completed items, current items, and future items, along with levels of payment.

The following is a breakdown of the study protocol items and their compensation rates: Baseline: $20, Social Network Interview 1: $25, Training: $40, Incentive for completing training within two weeks: $10, 3-Month Survey: $20, 6-Month Survey: $20, 9-Month Survey: $20, 12-Month Survey: $20, Bonus for completing all 4 surveys: $5, Social Network Interview 2: $25, Qualitative Personal Network Interview: $30, Weekly Text Survey (24 total): $4 each, Bi-Weekly Text Survey (12 total): $4 each, 6-Month Follow-Up Specimen Completion Incentive: $15, 12-Month Follow-Up Specimen Completion Incentive: $15, Maximum Completion Bonus for completing all study activities: $10.”
